# Optimization of Platinum Nanoparticles (PtNPs) Synthesis by Acid Phosphatase Mediated Eco-Benign Combined with Photocatalytic and Bioactivity Assessments

**DOI:** 10.3390/nano12071079

**Published:** 2022-03-25

**Authors:** Khalil ur Rehman, Mostafa Gouda, Umber Zaman, Kamran Tahir, Shahid Ullah Khan, Sumbul Saeed, Ebtihal Khojah, Alaa El-Beltagy, Ahmed A. Zaky, Mohamed Naeem, Muhammad Imran Khan, Noor Saeed Khattak

**Affiliations:** 1 Institute of Chemical Sciences, Gomal University, Dera Ismail Khan 29050, Pakistan; rehmankhalil025@gmail.com (K.u.R.); umberzaman25@gmail.com (U.Z.); tahirkamrangomal@gmail.com (K.T.); 2 College of Biosystems Engineering and Food Science, Zhejiang University, Hangzhou 310058, China; 3 Department of Nutrition and Food Science, Food Industries and Nutrition Research Institute, National Research Centre, Giza 12422, Egypt; 4 Department of Biochemistry, Women Medical and Dental College, Abbottabad 22080, Pakistan; 5 National Key Laboratory of Crop Genetic Improvement, Huazhong Agricultural University, Wuhan 430070, China; sumbulsaeed@webmail.hzau.edu.cn; 6 Department of Food Science and Nutrition, College of Science, Taif University, P.O. Box 11099, Taif 21944, Saudi Arabia; eyykhojah@gmail.com (E.K.); elbeltagy_alaa@yahoo.com (A.E.-B.); 7 Department of Food Technology, Food Industries and Nutrition Research Institute, National Research Centre, Giza 12422, Egypt; dr.a.alaaeldin2012@gmail.com; 8 Nutrition and Food Science of Ain Shams University Specialized Hospital, Ain Shams University, Cairo 11566, Egypt; naeem70.ash@gmail.com; 9 Department of Biomedical Sciences, Pak-Austria Fachhochschule, Institute of Applied Sciences and Technology, Mang Haripur 22620, Pakistan; muhammad.i.khan@gmail.com; 10 Center for Materials Science, Islamia College University, Peshawar 25120, Pakistan; noorsaeed24@yahoo.com

**Keywords:** acid phosphatase, PtNPs, photocatalytic activity, antibacterial activity, DPPH radicals

## Abstract

Noble metal nanoparticles (NMNPs) are viable alternative green sources compared to the chemical available methods in several approach like Food, medical, biotechnology, and textile industries. The biological synthesis of platinum nanoparticles (PtNPs), as a strong photocatalytic agent, has proved as more effective and safer method. In this study, PtNPs were synthesized at four different temperatures (25 °C, 50 °C, 70 °C, and 100 °C). PtNPs synthesized at 100 °C were smaller and exhibited spherical morphology with a high degree of dispersion. A series of physicochemical characterizations were applied to investigate the synthesis, particle size, crystalline nature, and surface morphology of PtNPs. The biosynthesized PtNPs were tested for the photodegradation of methylene blue (MB) under visible light irradiations. The results showed that PtNPs exhibited remarkable photocatalytic activity by degrading 98% of MB only in 40 min. The acid phosphatase mediated PtNPs showed strong bacterial inhibition efficiency against *S. aureus* and *E. coli*. Furthermore, it showed high antioxidant activity (88%) against 1,1-diphenyl-2-picryl-hydrazil (DPPH). In conclusion, this study provided an overview of the applications of PtNPs in food chemistry, biotechnology, and textile industries for the deterioration of the natural and synthetic dyes and its potential application in the suppression of pathogenic microbes of the biological systems. Thus, it could be used as a novel approach in the food microbiology, biomedical and environmental applications.

## 1. Introduction

Nowadays, inorganic nanoparticles are an integral cornerstone that reflects tremendous scientific and technical development [1,2,3,4]. NMNPs such as Ag, Au, Pd, and Pt have been highlighted to have several applications in material science, chemistry, and medicine [5,6,7]. Platinum nanoparticles (PtNPs) have received much more attention among researchers due to their distinctive structural, catalytic, and optical properties, as well as their large surface area, which makes them a promising choice for catalytic and biological applications [8,9]. PtNPs have been reported to be a capable and effective drug carrier [10]. PtNPs also cover a vast diversity of therapeutic properties, such as antifungal, antibacterial, anti-diabetic, and anticancer applications [11].

Recently, green chemistry has become progressively essential to the fabrication of eco-benign materials [12,13,14]. The increasing demand for innocuous, efficient, eco-benign, and green approaches has gained the interest of researchers around the world towards the green synthesis of nanoparticles [15]. Plants are preferred over other biosynthetic techniques as they do not require bacterial and fungal culturing or long-term conservation. Plant sources were also highlighted to be very selective and effective in manipulating the size, and dispersion of nanoparticles [16]. As a result, using plant extracts for nanoparticles synthesis promotes green chemistry concepts such as less harmful chemical syntheses, non-toxic solvents, energy-efficient design, and the utilization of sustainable resources.

Plant components such as roots, seeds, fruits, leaves, and stems, were also used to synthesize NPs due to their extract containing biomolecules (i.e., acid phosphatase, phenolics), which have a significant role in the stabilization and reduction in nanoparticles [17]. Plants are used in the manufacturing of PtNPs as they are simple, cheap, and eco-benign in comparison to other traditional approaches, and they produce more stable synthetic particles [18]. Plant extracts such as Crocus sativus, Tragia involucrate, and Nymphaea alba have recently been used in the synthesis of PtNPs [19,20,21].

The emission of toxic dyes into aquatic systems is a significant environmental problem [22,23,24,25,26,27]. A typical hazardous dye, methylene blue (MB), prevents sunlight from reaching the water body, causing long-term harm to the aquatic systems [28]. Therefore, this harmful dye was used to assess the photocatalytic activity of the as-synthesized PtNPs.

Microorganisms are considered to be concerned with several life-threatening infections both in humans and animals [29]. The *E. coli* and *S. aureus* are often found in food, environment, and humans as well as animal intestines [30], and can cause serious skin infections, respiratory and excretory tract issues [31]. Different antibiotics were used to treat these harmful bacteria, however, their continued use or intake led to the formation of drug-resistant bacteria [32]. Thus, innovative materials such as PtNPs must be prepared and used for this challenging task. Furthermore, biogenic PtNPs have been proved to exhibit potent antifungal activity against several fungal species [33]. Methods for determining the efficiency of nanoparticles like antioxidants have become more widely used. A typical way for investigating nanoparticles as antioxidants is the utilization of stable DPPH free radicals [34].

In this contribution, acid phosphatase of *C. intybus* seeds were used for the first time for the biosynthesis of PtNPs as an efficient and green synthetic approach. Acid phosphatase has performed an effective role in the formation and stabilization of PtNPs. The biogenic PtNPs were tested for the photocatalytic degradation of MB. Furthermore, PtNPs were also evaluated for their biomedical, i.e., antibacterial and antioxidant, applications.

## 2. Materials and Methods

### 2.1. Materials

Hexachloroplatinic (IV) acid (H2PtCl6), 2,7-dichlorodihydrofluorescien diacetate (DCFH-DA), nutrient agar, nutrient broth, and streptomycin were purchased from Sigma-Aldrich, (North Nazimabad, Pakistan). Inorganic phosphate (Pi), adenosine 5-triphosphate, para-nitrophenyl phosphate (p-NPP), sodium phytate, nicotinamide adenine dinucleotide phosphate (NADP), G-1-P (glucose-1-PO4) fructose-1-phosphate (F-1-PO4), and glucose-6-phosphate, were purchased from Sigma-Aldrich (Darmstadt, Germany). CM-cellulose, Con-A-4B-Sepharose, and Sephadex G-100 were procured from Pharmacia Biotech (Stockholm, Sweden). Sodium dodecyl sulfate-polyacrylamide gel electrophoresis and proteins marker were collected from Bio-Rad (Paris, France) and Sigma-Aldrich (Darmstadt, Germany). The whole experiment was carried out in deionized water.

### 2.2. Extraction of Acid Phosphatase Enzyme

Fresh seeds of C. intybus were taken from a local seeds corporation of Dera Ismail Khan city (Khyber Pakhtunkhwa, Pakistan). The seeds were crushed into a fine powder, and the crude enzyme was extracted by stirring the seed powder in a 10 mM Tris HCl buffer pH 7.4 (1 g seed powder/10 mL) for 60 min. After filtration with a cotton sheet, the supernatant was centrifuged for 20 min at 6000 rpm. In the acid phosphatase assay, the supernatants were used as a crude enzyme solution. The methods used for the extraction and purification of acid phosphatase were performed at 4 °C with slight modifications from Zaman et al. [35].

### 2.3. Synthesis of PtNPs Using Acid Phosphatase

To prepare PtNPs, 10 mL acid phosphatase was mixed with the 40 mL of (0.01 M) H2PtCl6 solution. The suspension was subjected to magnetic stirring for 80 min at four different temperatures (i.e., 25 °C, 50 °C, 70 °C, and 100 °C). The formation of PtNPs was indicated by the appearance of brown color. Thus, the suspension was centrifuged (3500 rpm, 5 min) and then dried in a freeze mobile 6ES freeze drier.

### 2.4. Photocatalytic Activity of PtNPs

The photocatalytic efficiency of acid phosphatase mediated PtNPs was assessed by the degradation of MB solution in the presence of visible light [36]. A 500 W tungsten filament bulb was used as a visible light source. According to the procedure, 8 mg of PtNPs prepared at optimized conditions (i.e., 25 °C, 50 °C, 70 °C, and 100 °C) were added to 80 mL (16 mg/L) of MB solution. To accomplished dye-catalyst equilibrium, the solution was placed at room temperature under visible light with continuous stirring. The progress of reactions was observed using a UV-visible spectrophotometer (Shimadzu-2400) after regular intervals of time. The same experimental conditions without nanoparticles were used as a negative control. The below formula was applied to calculate the percentage of photocatalytic degradation activity:

$$\%\;\mathrm{Degradation}=\frac{A_{o}-A_{t}}{A_{o}}\times 100$$

where (*A_o_*) represents the initial absorbance of MB at time zero, while (*A_t_*) refers to the absorbance of MB at specific time.

### 2.5. Antibacterial Test

The antibacterial activity of acid phosphatase mediated PtNPs was tested by agar well diffusion protocol using *E. coli* and *S. aureus* as tested bacteria [37]. This method has been applied to examine the range, to which bacteria have been affected by nanoparticles. The bacteria were grown in an agar medium at 37 °C for 24 h The bacteria were inoculated into Muller–Hinton agar plates through sterilized swabs [38]. Bores of 6 mm diameter were formed in Petri dishes with a disinfected cork drill. After that, 1 mg of PtNPs were added into 1 mL of water under visible light for 80 min and then properly poured 50 μL of PtNPs were added into the wells. Finally, the Petri dishes were placed in an incubator for 24 h at 37 °C. Streptomycin was used as a standard in this assay.

#### 2.5.1. Minimum Inhibitory Concentration (MIC)

The MIC is the least quantity of a substance that inhibits the growth of bacteria. A serial dilution protocol was followed for determining the MIC of PtNPs [39]. Next, 1 mL of each bacterial solution was poured into the ultra-clean test tubes. Then different concentrations (10–80 µg/mL) of PtNPs were introduced into the tubes. Subsequently, the test tubes were homogenized, put on sanitized petri dishes, and incubated for 24 h at 37 °C. The test tubes with only growth media were used as a negative control. The assay was repeated three times.

#### 2.5.2. Reactive Oxygen Species (ROS) Production

The intracellular formation of ROS is regarded to be the key factor in the bacterial therapy of metal-based nanoparticles. The intracellular formation of ROS by as-prepared NPs was discovered through a simple experiment. The gram-negative *E. coli* strain was cultured in LB media and then treated with PtNPs before being placed in the dark for 4 h at 37 °C. The treated samples were cleaned with Phosphate Buffered Saline (PBS, pH 7.4) and then 20 mM of 2,7-dichlorodihydrofluoresceindiacetate (DCFHDA) was added dropwise. After that, fluorescent dye-treated bacterial cell samples were centrifuged (8000 rpm, 10 min) and washed thrice to eradicate the extracellular dye. The fluorescence images were obtained using a fluorescence microscope with wavelengths of 488 nm and 535 nm for excitation and emission, respectively [40].

### 2.6. Antioxidant Activity of PtNPs

The antioxidant activity was measured by using 1,1-diphenyl-2-picryl-hydrazil (DPPH) radical scavenging assay for acid phosphatase mediated PtNPs following Khan, et al. [41], with some modifications. In brief, 0.5 mL (1 mM DPPH) was continuously agitated in the dark for 20 min with different concentrations (0–1 mg/mL) of PtNPs. After incubation, the absorbance was measured through a spectrophotometer at 517 nm against methanol (control). Vitamin C was used as a standard in this study. The percentage of inhibition was calculated using the following formula.

$$\%\;\mathrm{Inhibition}=\frac{\mathrm{Absorbance}\;\left(\mathrm{Control}\right)-\mathrm{Absorbance}\;\left(\mathrm{Test}\right)}{\mathrm{Absorbance}\;\left(\mathrm{Control}\right)}\times 100$$

### 2.7. Surface Characterization

The formation of biosynthesized PtNPs was confirmed by various microscopic and spectroscopic techniques. UV–visible spectrophotometer (Shimadzu 2400, Tokyo, Japan) was used for the determination of surface plasmon resonance peak (SPR) and optical properties. An ABB MB3000 spectrophotometer (StellarNet, Inc., Tampa, FL, USA) was used for the measurements of FT-IR analysis. The crystalline nature of PtNPs was determined by using a Rigaku D/Max 2500 VB+/PC diffractometer. XPS was recorded through a VG Scientific ESCA Lab220i-XL spectrometer with Al Kα radiation in twin anode at 14 kV × 16 Ma, calibrated with confinement carbon (C 1s 284.6 eV). Nano-ZS instrument (HORIBA Zetasizer SZ100, Worcestershire, UK). was employed to analyze the size of the NPs. Furthermore, the surface morphology and microstructure of PtNPs were evaluated using scanning electron microscopy (SEM, Hitachi S-4700, Tokyo, Japan). In brief, samples were freeze–dried and then put on the microscope port before scanning was performed at a temperature below 110 K and at an accelerating voltage of 100 kV. For the high resolution transmission electron microscopy (HREM), JEOL JEM-4000EX microscope (HRTEM, JEM-3010, Milpitas, CY, Canada) was used at 400 kV with a point to point resolution of about 0.17 nm. The elemental composition and distribution maps of PtNPs were investigated using energy-dispersive X-ray spectroscopy (EDX) with a JEOL Scanning Electron Microscope Model JSM-5910 (Tokyo, Japan) with its imaging capability to identify the specimen of interest. Samples with about 50 mm diameter (20 mm thick) were accommodated in the X-ray chamber of the machine. Spectra of the expected elements were obtained with detection limits of ~0.7 weight percent.

## 3. Results and Discussion

### 3.1. XRD Analysis

**Figure 1:**
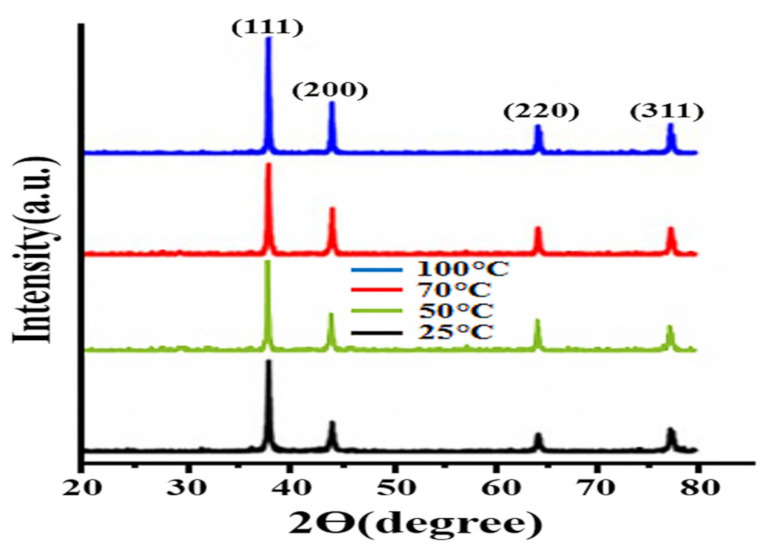
XRD pattern of PtNPs synthesized at 25 °C, 50 °C, 70 °C and 100 °C, respectively.

X-ray diffraction analysis was carried out to determine the crystalline structure of PtNPs. The XRD spectra of PtNPs synthesized at optimized conditions are shown in Figure 1. The 2θ range was adjusted between 20–80°. The results clearly showed that four distinct peaks were observed at 38.1°, 44.6° 64.7° and 78.3° associated with the (111), (200), (220) and (310) lattice planes of the f.c.c. crystal structure of PtNPs [42]. The data obtained are in agreement with the JCPDS File No. 04-0783 and 04-0802. Furthermore, the (111) plane was the preferred growth orientation of the biosynthesized PtNPs. The Scherer formula was used to calculate the average size of nanoparticles synthesized at optimized conditions, which were found to be 2, 2.5, 3, and 4 nm, respectively. Additionally, the smaller crystal size reported in this work indicates that the synthesized PtNPs have a higher proficiency.

### 3.2. FT-IR Analysis

**Figure 2:**
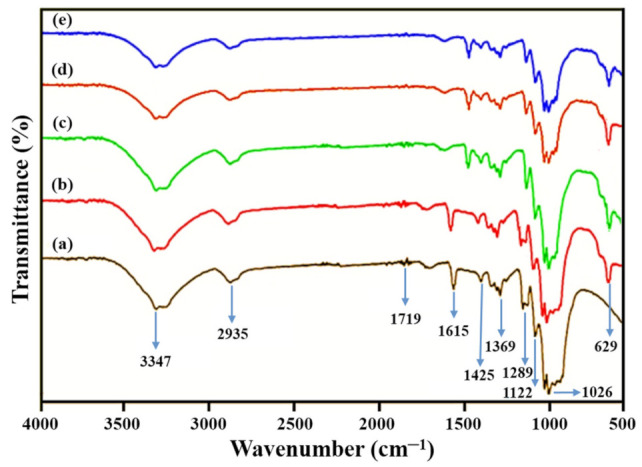
(**a**) FT-IR spectra of Acid phosphatase and (**b**–**e**) PtNPs synthesized at 25 °C, 50 °C, 75 °C and 100 °C respectively.

FT-IR analysis is quite functional to define the structural features and nature of biosynthesized nanoparticles. The FT-IR spectra of acid phosphatase and PtNPs are illustrated in Figure 2. The spectra obtained displayed some prominent bands at 3347 cm^−1^, 2935 cm^−1^, 1719 cm^−1^, 1615 cm^−1^, 1425 cm^−1^, 1369 cm^−1^, 1289 cm^−1^, 1122 cm^−1^, 1026 cm^−1^ and 629 cm^−1^. The broad bands that appeared at 3347 cm^−1^ and 1289 cm^−1^ correspond to N–H bonds [43]. The bands obtained at 2935 cm^−1^ and 1615 cm^−1^ reflect the stretching and bending vibration of O–H bonds, respectively [44]. The bands at 1719 cm^−1^ are related to the -COOH group [45]. The bands at 1425 cm^−1^ and 1369 cm^−1^ are associated with the C–N bonds [46]. The appearance of bands at 1122 cm^−1^ and 1026 cm^−1^ are due to bending vibrations of C–C and C–O bonds, respectively [47]. The arrival of a new band at 629 cm^−1^ indicated the formation of PtNPs.

### 3.3. UV-Visible Analysis

**Figure 3:**
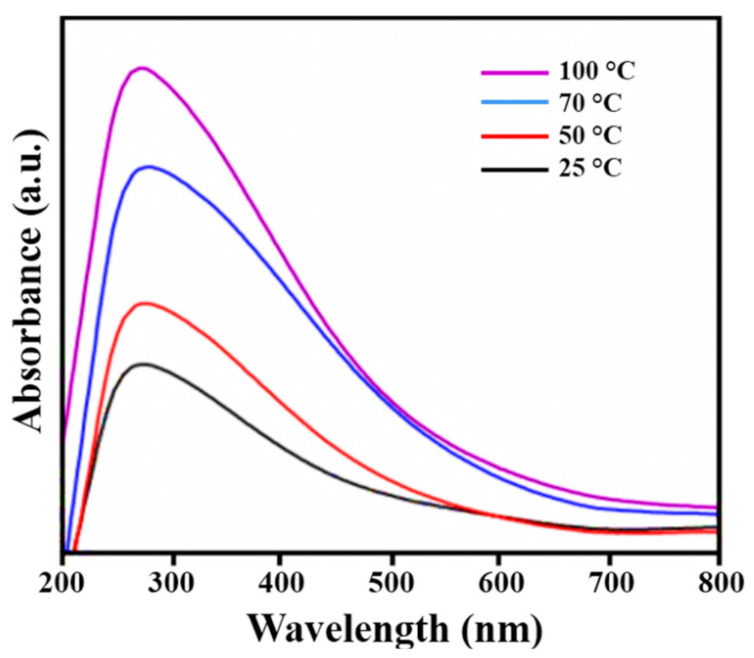
UV-Vis analysis of PtNPs synthesized at 25 °C, 50 °C, 70 °C, and 100 °C, respectively.

UV-Visible analysis was primarily applied to authenticate the formation of PtNPs. Figure 3 depicts the UV-visible absorption spectra of acid phosphatase-mediated PtNPs. Surface plasmon resonance (SPR) is the excitation produced by a light source at a certain wavelength, as determined by UV–visible spectroscopy [48]. Most of MNPs exhibited characteristic SPR bands [49]. Furthermore, the nature and location of the SPR peak are usually based on the size and morphology of NPs. The SPR peaks for PtNPs were examined at four different reaction temperatures (i.e., 25 °C, 50 °C, 70 °C, and 100 °C). The peak at 275 nm confirmed the development of PtNPs [10,47,48]. The SPR peak intensities were varied with the change of temperature [50]. Low intense peaks were observed at 25 °C and 50 °C, which may be due to the excitation energy of platinum valance band electrons. Smaller contents of PtNPs were formed that were confirmed by HRTEM analysis. Whereas, highly intense peaks were obtained at 70 °C and 100 °C, which may be due to high content and smaller sized PtNPs [51].

### 3.4. HRTEM, SAED and DLS Analysis

**Figure 4:**
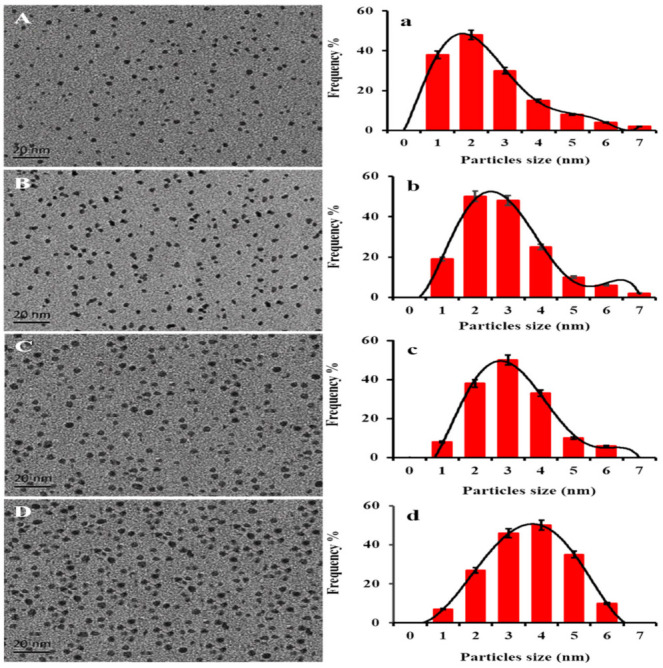
HRTEM images (**A**–**D**) and particle size distribution (**a**–**d**) of PtNPs synthesized at 100 °C (**A**), 70 °C (**B**), 50 °C (**C**), and 25 °C (**D**), respectively.

High Resolution Transmission Electron Microscopy (HRTEM) is one of the most advanced techniques used to evaluate the size, shape, and dispersion of nanoscale materials [52]. In this study, the HRTEM micrographs of PtNPs synthesized at the optimized conditions are shown in Figure 4A–D. Furthermore, the DLS method was used to measure the hydrodynamic size [53]. Figure 4a–d represents the DLS examinations of PtNPs synthesized at 25 °C, 50 °C, 70 °C, and 100 °C, respectively. The results showed that the particles obtained at 25 °C and 50 °C had several aggregates and the biggest size, with a frequency 54 ± 6% at 4 nm and 55 ± 3% at 3 nm compared to the 100 °C with 54 ± 2% at 2 nm (Figure 4A–D,a–d). PtNPs synthesized at 70 °C also had a bigger size than 100 °C with spherical shapes and uniform dispersion. Whereas, PtNPs synthesized at 100 °C had the smallest size, and sphere morphology with a high degree of dispersion.

The results indicated that 100 °C is the best temperature for the bio-fabrication of PtNPs. The reason was, at 100 °C the particles were uniformly distributed and smaller in size than those obtained at 70 °C, 50 °C and 25 °C. Nishanthi, et al. [54] mentioned that the increase in temperature is considered to be the most significant factor regarding the direct decrease in the metal nanoparticles size, which allows their shape to be sharper, which in turn can increase their antimicrobial and other bioactivities. The prior studies also supported the HRTEM results of biosynthesized PtNPs [54]. The hydronic sizes were found to be 2, 2.5, 3, and 4 nm. The hydrodynamic particle size is greater than the HRTEM particle size as it contains the size of acid phosphatase that caps the nanoparticles [55], in which, the most prevalent morphology was spherical. Moreover, the selected area electron diffraction (SAED) analysis was performed to determine the crystalline structure of PtNPs. The typical SAED pattern of PtNPs was depicted in Figure 7A. The SAED results matched the XRD data, which confirmed the (111), (200), (220), and (311) planes of crystalline PtNPs [56].

### 3.5. X-ray Photoelectron Spectroscopic (XPS) Analysis

**Figure 5:**
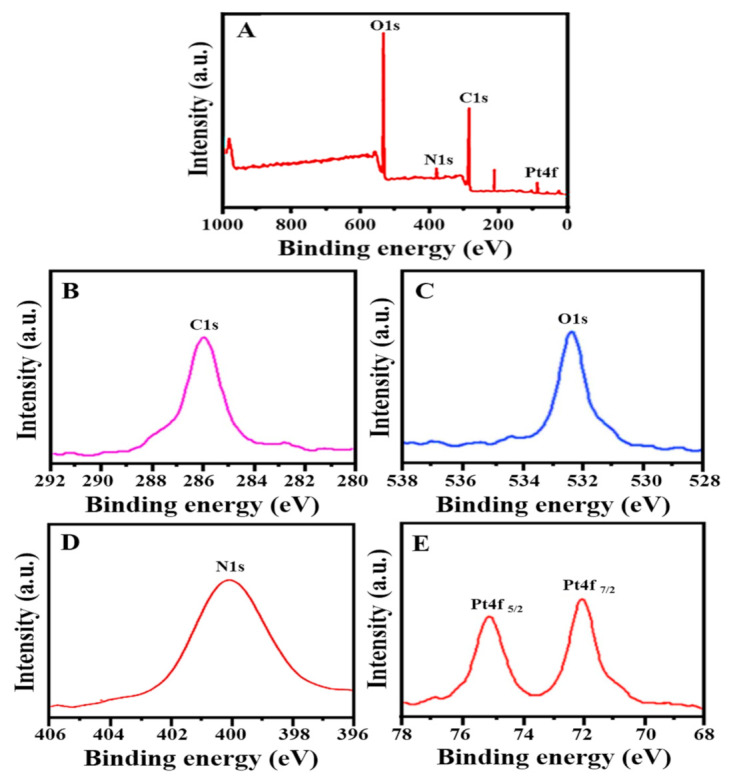
XPS spectrum of PtNPs (**A**) survey, (**B**) C1s, (**C**) O1s, (**D**) N1s and (**E**) Pt4f.

X-ray photoelectron spectroscopic technique was applied to analyze the elements and valance state of PtNPs. The most common elements in our findings were Pt, C, O, and N as shown in Figure 5A. Figure 5B,C demonstrated that the high-resolution spectra of C1s and O1s exhibited main peaks with binding energies of 286.08 eV and 532.48 eV, respectively, where C and O elements came from acid phosphatase [57,58,59]. Figure 5D exhibited a peak at 399.78 eV, which corresponds to N1s core levels. Figure 5E indicated that the high-resolution spectra of Pt4f were typical for metallic Pt, with binding energies of 72.48 and 75.58 eV for Pt^0^ 4f7/2 and Pt^0^ 4f5/2, respectively. The obtained results were similar to previously reported studies [59]. As a result, rather than Pt^2+^ or Pt^4+^, PtNPs were found to be in the metallic Pt^0^ form (only the zero-valent state is obtained). The obtained result could be explained by acid phosphatase obtained from C. intybus seeds simply reducing an oxidized platinum species to its zero-valent form (the stable state of Pt).

### 3.6. SEM and EDX Analysis

**Figure 6:**
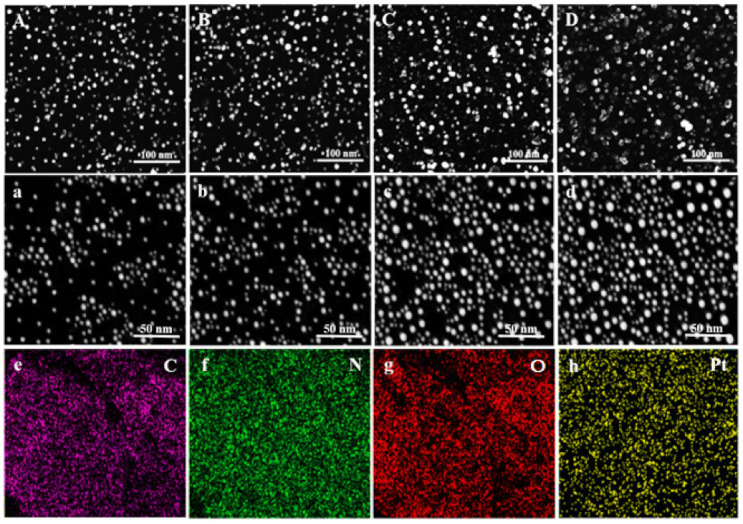
Represents the SEM micrographs of PtNPs synthesized (**A**–**D**; 100 nm) and (**a**–**d**; 50 nm) at 100 °C, 70 °C, 50 °C, and 25 °C, respectively; while (**e**–**h**) represent the EDS mapping of C, N, O and PtNPs, respectively.

The size, shape, and surface morphology of nanoparticles were investigated using SEM analysis. The SEM micrographs of PtNPs prepared at 100 °C, 70 °C, 50 °C, and 25 °C, are illustrated in Figure 6A–D,a–d, respectively. The SEM images were obtained at different magnifications. According to SEM results, the average size of PtNPs is found to be 2 nm, 2.5 nm, 3 nm and 4 nm, respectively. The white dots represent PtNPs, which have a small size, spherical shape, and are uniformly dispersed. These results were similar to the findings of Ali and Mohammed [60].

**Figure 7:**
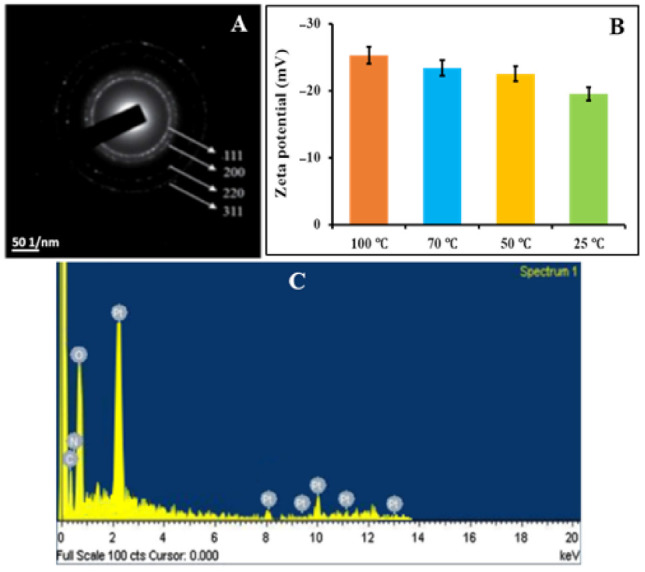
(**A**) SAED pattern, (**B**) Zeta potential analysis and (**C**) EDS spectrum of PtNPs.

The bio-reduction in PtNPs was further confirmed through energy dispersive spectroscopy (EDS). The EDS spectrum of biocapped PtNPs is shown in Figure 7C. The development of PtNPs was confirmed by the presence of strong peaks of elemental Pt at energy levels of 2.2, 8.2, 9.5, 10, 11.2, and 13 KeV. The results are consistent with the reported previous studies [61]. The EDX profile clearly showed that the weak signals like C, O, and N may have originated from biomolecules (i.e., acid phosphatase) present on the surface of nanoparticles [62]. These organic compounds performed a substantial role in the capping, stabilization, and formation of PtNPs. Furthermore, the corresponding elemental mapping images showed that all elements (C, N, O, and Pt) are present in the sample, confirming the successful synthesis of PtNPs. The EDS mapping of acid phosphatase-mediated PtNPs is shown in Figure 6e–h.

### 3.7. Zeta Potential Analysis

Zeta potential analysis was used to investigate the surface charge and stability of PtNPs. This method is often used to analyze the dispersion stability of nanoparticles [63]. PtNPs synthesized at 100 °C and 70 °C had zeta potential values −25.3 mV and −23.4 mV with higher stability than 50 °C and 25 °C with −22.5 mV, and −19.5 mV, respectively (Figure 7B). Shah, et al. [64] mentioned that nanoparticles becme more stable as their zeta potential value becomes more negative. Furthermore, the acid phosphatase-mediated PtNPs have a negative zeta potential, indicating that they are capped with negatively charged biomolecules that cause repulsion (dispersion) among them and boost their stability. The current findings were lower than those of Ullah, et al. [65], who reported a zeta potential of −41 mV for PtNPs, and higher than those of Nishanthi, et al. [66], who reported a zeta potential of −13 mV for PtNPs.

### 3.8. Applications of PtNPs

#### 3.8.1. Photocatalytic Degradation of MB by PtNPs

**Figure 8:**
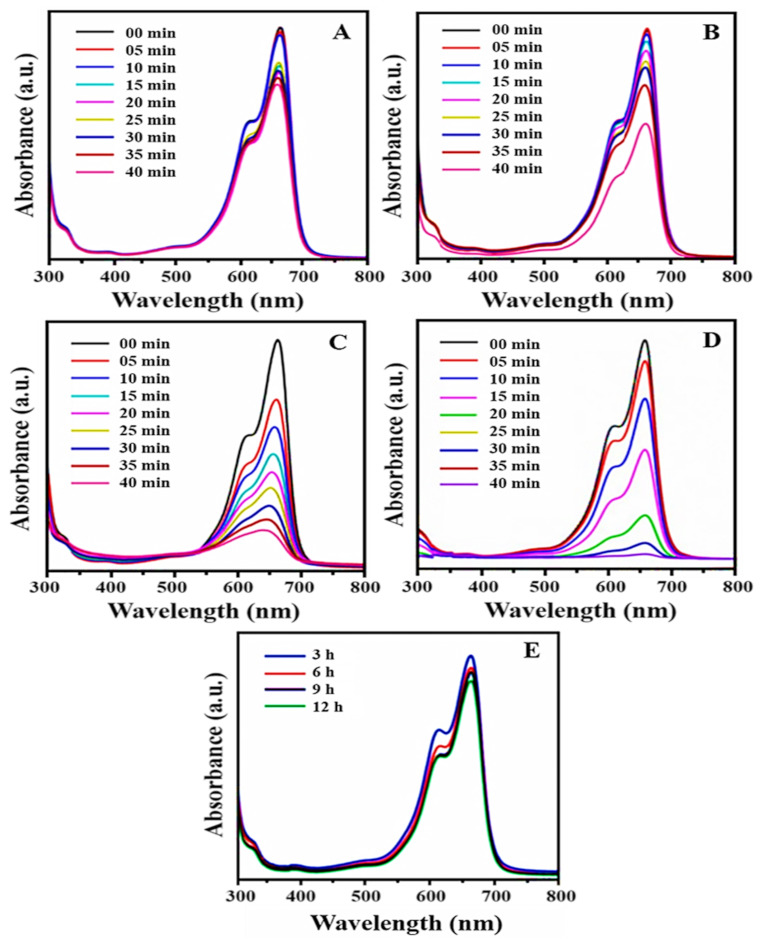
Photocatalytic degradation of MB in the presence of PtNPs synthesized at (**A**) 25 °C, (**B**) 50 °C, (**C**) 70 °C, (**D**) 100 °C and (**E**) photodegradation of MB in the absence of PtNPs.

**Figure 9:**
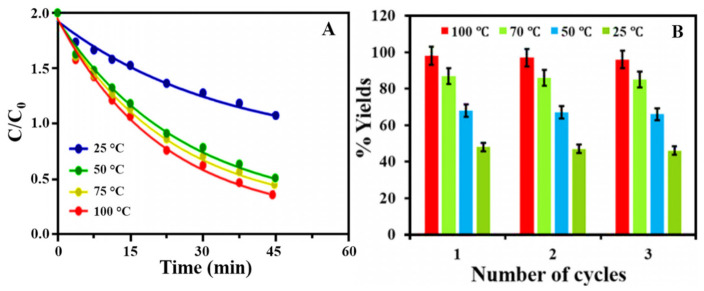
(**A**) plot of (C/C_0_) versus time for the catalytic degradation of MB in the presence of PtNPs synthesized at 25 °C, 50 °C, 70 °C and 100 °C, respectively, and (**B**) Reusability of PtNPs for the degradation of MB.

MB has been identified as the major source of industrial water pollution, with numerous harmful effects on human health that must be eliminated from effluents. In the present investigation, the photocatalytic activity of biosynthesized PtNPs was examined by the degradation of MB under visible light irradiations. Generally, the maximum absorption band corresponding to MB was observed at 664 nm [67,68]. The addition of PtNPs to MB solution resulted in a significant reduction in absorption peaks, indicating MB degradation. Figure 8 symbolizes the UV–Vis absorption of MB degradation in the presence of PtNPs synthesized at 25 °C, 50 °C, 70 °C, and 100 °C, respectively. Figure 8A represents the photocatalytic degradation of MB in the presence of PtNPs synthesized at 25 °C. Figure 8B represents the photodegradation of MB in the presence of PtNPs synthesized at 50 °C, while Figure 8C depicts the photodegradation of MB in the presence of PtNPs synthesized at 70 °C and Figure 8D describe the photodegradation of MB in the presence of PtNPs synthesized at 100 °C. The results clearly show the photocatalytic efficiency of PtNPs by degrading 48%, 67%, 86%, and 98% of MB, respectively, after 40 min of irradiations. In particular, PtNPs synthesized at 100 °C exhibit superior photocatalytic activity of 98% degradation within 40 min. This shows an outstanding percentage of degradation as compared to PtNPs synthesized at 70 °C (86%), 50 °C (67%), and 25 °C (48%). This remarkable efficiency of PtNPs is due to their trivial size, and spherical morphology with a high degree of dispersion. The degradation of MB under visible light illumination in the absence of nanoparticles is shown in Figure 8E. The absorption peak of MB slowly decreased and reached a maximum degradation (11%) at 12 h. The results showed that in the absence of catalysts, MB concentration decreased slowly, which may be attributable to the self-photo fading phenomena of MB in the presence of light [69]. Figure 9A represents the photocatalytic degradation of MB in the presence of PtNPs as a plot of absorbance versus time. The kinetic study of MB degradation indicates that the reaction is a pseudo-first-order kinetic reaction. According to the results, the rate constants (k) for these reactions were found to be (k = 5.781 × 10^−3^ min^−1^, 5.534 × 10^−3^ min^−1^, 5.359 × 10^−3^ min^−1^, and 5.842 × 10^−2^ min^−1^), respectively, which indicate the high catalytic efficiency of PtNPs. The present study reveals that as-synthesized PtNPs have exceptional photocatalytic activity, enabling them to be used for large-scale MB degradation. Table 1 also includes a detailed comparison of PtNPs and other platinum nanoparticles, which comprises factors such as dye concentration, degradation efficiency, and time. Table 1 shows that the prepared PtNPs are very effective in the photocatalytic degradation of MB.

##### (1) Factors Affecting the Photocatalytic Activity of MB

Several factors, such as irradiation time, catalyst dosage, and initial dye concentration affect the photocatalytic degradation of MB. It is necessary to know which values of the aforementioned factors provide productive efficiency, and which parameters have the maximum effect concerning the degradation of MB.

##### (2) Effect of Irradiation Time

**Figure 10:**
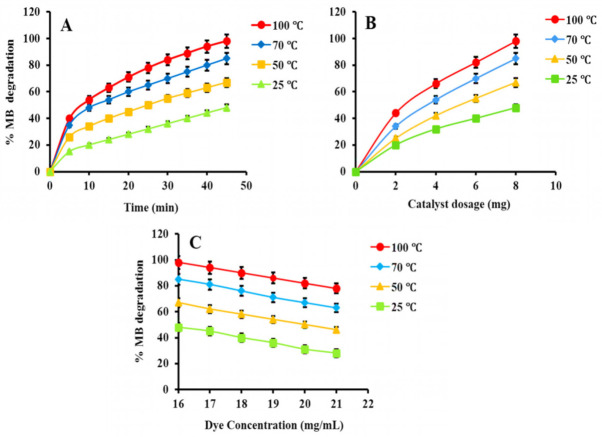
(**A**) Effect of irradiation time, (**B**) catalyst dosage and (**C**) initial dye concentration on the photocatalytic degradation of MB.

Light irradiation has a major effect on the photodegradation of MB. The photodegradation activity of PtNPs increases as the irradiation time increases. This is due to photoelectrons being formed when the valence electrons are excited by visible light. The photodegradation of MB is caused by •OH radicals produced by these highly energetic photoelectrons. To investigate the photodegradation efficiency of nanoparticles at different time intervals, the effect of irradiation time on the photodegradation of MB was examined. The assay was performed in the presence of light for 45 min at ambient temperature. Figure 10A shows a graph demonstrating the percentage of dye degradation vs. light irradiation. The results indicate that MB degradation is proportional to irradiation time. In the presence of PtNPs synthesized at 100 °C, 70 °C, 50 °C and 25 °C, 98%, 86%, 67%, and 48% of MB was degraded, respectively, in 45 min of irradiations. In the comparative analysis, PtNPs synthesized at 100 °C were found to be an excellent agent for MB degradation.

##### (3) Effect of Catalyst Dosage

The effect of catalyst dosage on MB degradation was examined by increasing the concentration of PtNPs from 2–8 mg. The dye concentration was fixed at 16 mg L^−1^. Figure 10B depicted a graph of the percentage of dye degradation versus time. The amount of catalyst has a direct relationship with photocatalytic efficiency. The results showed that increasing the PtNPs concentration from 2–8 mg L^−1^ increased the photo deprivation of MB (48–98%). This is the result of high concentrations of the nanoparticles producing maximum radicals [74,75].

##### (4) Effect of Dye Concentration

The photocatalytic degradation efficiency of PtNPs is also affected by the initial dye concentrations. The assay was performed in 80 mL water having MB from 16–21 mg L^−1^ and 8 mg PtNPs. The number of active sites on the surface of the catalyst can be used to control the photodegradation process. The photocatalytic efficiency is higher for less initial dye concentration as shown in Figure 10C. Furthermore, at higher concentrations, the dye serves as a barrier against light radiation, resulting in reduced photocatalytic efficacy [76].

##### (5) Reusability

The reusability test was carried out with the most effective PtNPs for MB degradation to ensure that the catalyst was stable sufficiently for practical applications. In this analysis, the recovered catalysts were washed several times with water and acetone following the photodegradation activity, then dried at 100 °C before being used in the next experiment. The reaction time was fixed for 30 min in each cycle, and the findings indicated no perceptible decrease in degrading efficiency even after three cycles, as shown in Figure 9B. The foregoing results indicate that the photocatalysts are quite stable, as it remains unchanged after three cycles.

#### 3.8.2. Antibacterial Activity

Nowadays, the bacterial infection is a major threat to society and an issue of concern in the pharmaceutical sector. Therefore, most pharmaceutical companies and research institutions are concentrating their attention on developing antibacterial drugs using natural resources. According to Ahmed et al. [77], green synthesized PtNPs have an appreciable antibacterial activity against several pathogenic microbes. PtNPs were tested for bacterial inhibition activity against *E. coli* and *S. aureus* in this analysis. The *S. aureus* is a common, highly virulent bacteria that can cause several infections such as osteomyelitis, endocarditis, and necrotizing pneumonia [78]. Similarly, *E. coli* also causes serious infections both in humans and animals [79]. PtNPs have much stronger bacterial inhibition activity than those reported previously [80]. Recently, these bacterial strains showed resistance to a wide range of antibiotics, transforming them into harmful bacteria. Hence, it is essential to search for an alternate biocompatible substance to control these bacteria. The photoactive PtNPs exhibited strong antibacterial activity under both dark and visible light conditions. The antibacterial activity of PtNPs was considerably enhanced by visible light irradiation and hindered their growth. Table 2 shows that the antibacterial efficacy of the synthesized nanoparticles is substantially higher in visible light than in dark.

**Figure 11:**
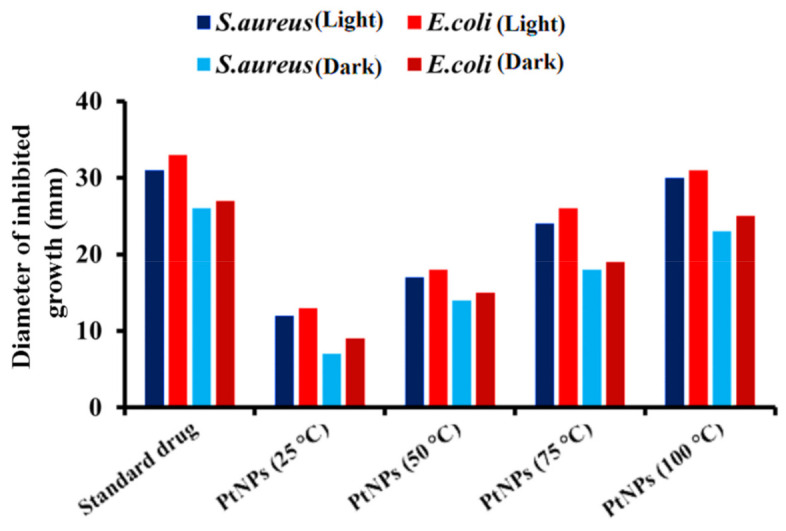
Zones of bacterial inhibition in the presence of standard drug and PtNPs synthesized at optimized conditions.

In this study, the biosynthesized PtNPs were found to significantly inhibit the growth of *E. coli* and *S. aureus* under visible light irradiation. However, the inhibition activity of PtNPs is relatively weaker in the absence of light as shown in Figure 11. PtNPs synthesized at 100 °C have a maximum zone of inhibition of 31 mm, 30 mm, 25 mm and 22 mm against *E. coli* and *S. aureus* under both sets of conditions, respectively. The remaining nanoparticles prepared at 70 °C, 50 °C, and 25 °C, have zones of inhibition, respectively, against *E. coli* and *S. aureus,* as shown in Table 2. According to these findings, we can conclude that the acid phosphates mediated PtNPs have excellent bacterial inhibition properties in the presence of visible light irradiations. The outer cell membrane of bacteria serves as their primary defense system. In general, metallic nanoparticles bind to the outer cell wall of bacteria, causing the lipoproteins/outer membrane to rupture, restricting cell division and ultimately leading to cell death [81,82,83].

PtNPs can cause an increase in ROS, which could also result in DNA downregulation, oxidative stress, and ultimately bacterial cell death [84]. PtNPs showed significant bacterial inhibition activity due to their trivial size, high distribution, and spherical morphology. Small size nanoparticles often have the greater surface area and are therefore more effective than those with bulk particle size. Antibacterial activity is commonly associated with the local activity of materials, which inhibits bacterial growth without causing tissue damage [85]. Researchers have also noted that it was not only the surface charge; the physicochemical properties of nanoparticles, like size, shape, and surface-volume relation, were also correlated to antibacterial activity [86]. Some researchers also suggested that negatively charged PtNPs can target Gram-negative bacteria through metal depletion [87]. As a result of the data collected, it was found that the newly synthesized PtNPs are potent antibacterial agents with outstanding efficiency towards Gram-negative bacteria at a concentration of 60 µg/mL. Table 3 shows a comparison of the antibacterial activity of PtNPs and other synthesized AgNPs.

#### (1) Mechanism of Bacterial Inhibition

**Figure 12:**
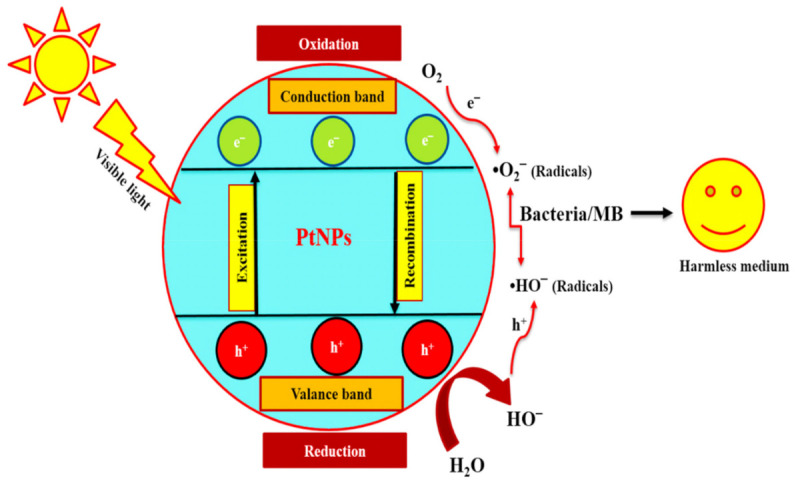
Schematic representations of ROS production in the presence of PtNPs.

It is very challenging to study the exact mechanism of bacterial inhibition activity in nanoparticles. So far, several mechanisms have been proposed for MNP-induced bacterial inhibition. In this contribution, we have tried to investigate the exact mechanism of bacterial suppression in the presence of PtNPs under visible light irradiation. The MNPs adhere to the bacteria cell wall, promoting the production of free radicals such as •O_2_^−^ and •OH radicals, which cause bacterial destruction [93]. Through respiratory burst activity, metal ions react to the thiol group of essential enzymes and denature them [94]. MNPs also interfere with nucleic acid (DNA), disrupting the cellular transport system. Figure 12 depicts the bacterial inhibition mechanism of PtNPs in the presence of visible light irradiations.

Figure 12 represents the proposed photocatalytic activity of PtNPs. Photons with energies greater than the bandgap energy are absorbed by the photocatalyst during the photocatalytic process, resulting in electron and hole pairs on the photocatalyst’s surface. These electrons and hole pairs can then be used in oxidation and reduction reactions, which often lead to the generation of ROS such as •OH and •O_2_^−^, which participate in dye degradation [69]. They produced ROS at the interface of photocatalysts and aqueous solution, increasing the oxidation and reduction reactions. The ability of PtNPs to generate ROS has been widely reported [95]. Mechanism-1 demonstrates the stepwise production of ROS in the presence of PtNPs under visible light irradiations. In this analysis, the formation of ROS was attributed to the photocatalytic activity of PtNPs in the degradation of MB. The excited electrons of PtNPs produce reactive oxygen intermediates, which enhance the formation of ROS. These ROS seem to accumulate on the surface of the MB and play an effective role in their decomposition.

##### (2) Generation of ROS

**Figure 13:**
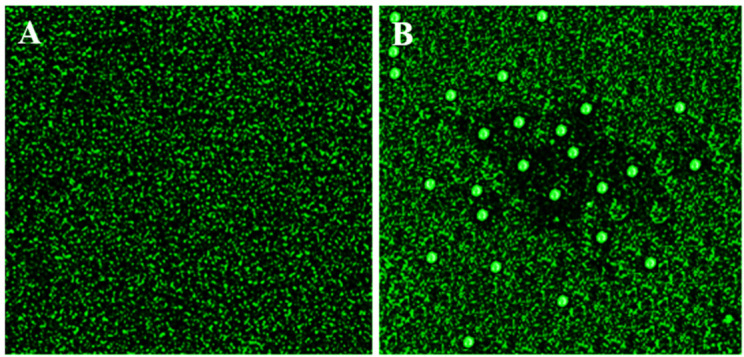
ROS analysis of *E. coli*. (**A**) in the absence and (**B**) in the presence of PtNPs.

The interaction of PtNPs with bacteria can result in the generation of ROS. The surface and excited electrons of PtNPs generate ROS (i.e., •OH, •O_2_^−^). Such ROS are involved in the oxidative stress and disintegration of proteins and DNA of bacteria. It is well known that DCFH-DA is a nonpolar dye that is oxidized by intracellular ROS to produce the polar derivative DCFH [96]. Figure 13A,B depicts the fluorescence images of the control and treated samples after feature extraction. There was almost no green color in the control sample as Illustrated in Figure 13A. However, *E. coli* treated with PtNPs showed a considerable increase in green fluorescence, indicating that PtNPs formed a significant proportion of ROS as illustrated in Figure 13B. These findings suggested that excited electrons of PtNPs cause oxidative stress, which is correlated to interactions with bacteria’s cell organelles (i.e., DNA), that eventually lead to cell death.

##### (3) Effect of PtNPs on the Surface Morphology of *E. coli* Cell

**Figure 14:**
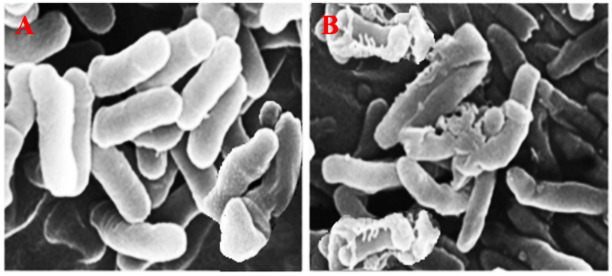
SEM examination of *E.coli* (**A**) in the absence and (**B**) in the presence of PtNPs.

The ultra-fine structure of *E. coli* cells mixed with bio-directed PtNPs was also examined using scanning electron microscopy. PtNPs in phosphate buffer saline solution (0.06 M, pH 7) were used to preserve *E. coli* cells, and then subsequently incubated for 3 h at 37 °C. Bacterial solutions without NPs was used as a control. The *E. coli* cells were collected and immersed again in PBS solution after incubation. Subsequently, a droplet of bacterial suspension was placed on silica glass with 2.5% glutaraldehyde and preserved at 4 °C for 18 h. Finally, the *E. coli* cells were washed thrice with ethanol and then inspected through a scanning electron microscope. The impact of PtNPs on the surface morphology of bacteria is shown in Figure 14A,B. The results show that when *E. coli* cells were mixed with PtNPs, they become denatured, shrink, and eventually die.

##### (4) Determination of MIC

The MIC value can be used to assess the efficiency of nanoparticles. MIC is the lowest inhibitory concentration of nanoparticles used to inhibit the growth of bacteria. A serial dilution protocol was followed for the determination of MIC. Mixing of dilute suspensions of PtNPs with *E. coli* and *S. aureus* solutions in artificial media can be used to quantify the antibacterial activity of PtNPs synthesized at optimized conditions (i.e., 25 °C, 50 °C, 70 °C, and 100 °C). The activity can be focused based on the zone of inhibition after a specific period of incubation. The MIC value for this activity was 30, 20, 20, and 10 μg mL^−1^, respectively, for both *E. coli* and *S. aureus*, as shown in Table 4, which are considered excellent inhibitory concentrations.

##### (5) Efficacy of PtNPs with Irradiation Time

**Figure 15:**
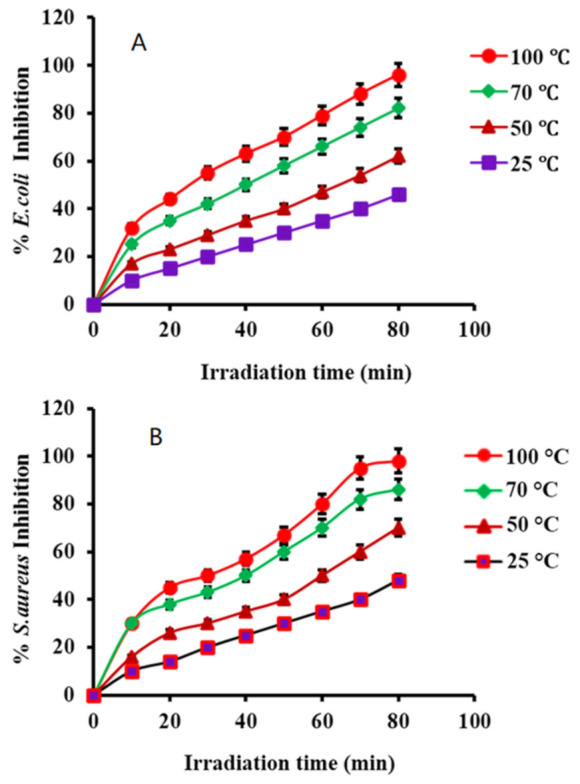
Irradiation effect on the inhibition of (**A**) *E. coli* and (**B**) *S. aureus* in the presence of PtNPs.

The catalytic efficiency of PtNPs was investigated at different irradiation intervals at room temperature. According to our results, bacterial inhibition increased with irradiation time. By irradiating the synthesized NPs with light, electrons will be excited, making the inhibition process much easier. The excited electrons generate radicals such as •O_2_^−^ and •OH, which kill the bacteria. The percent inhibition of bacteria increased proportionally as the irradiation time was increased. According to a time-dependent inhibition analysis, 96% of the bacteria were destroyed in the presence of PtNPs after 80 min of irradiation (see Figure 15A,B). This study showed that PtNPs were found to be more effective antibacterial agents.

##### (6) Concentration Effect of PtNPs

**Figure 16:**
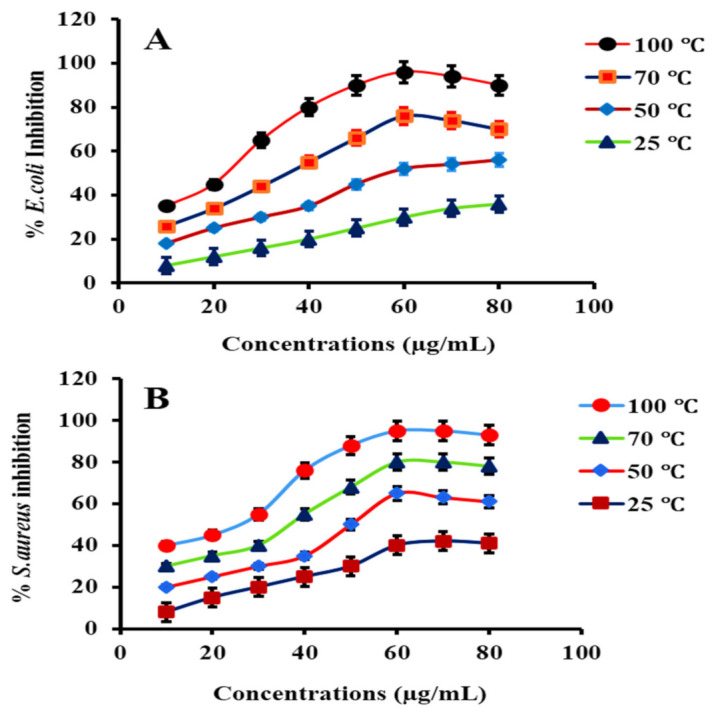
Concentration effect on the inhibition of (**A**) *E. coli* and (**B**) *S. aureus* in the presence of PtNPs.

The photoinhibition efficiency of biosynthesized PtNPs was assessed at different concentrations (10–80 μg/mL) of nanoparticles. The sample was irradiated for 80 min before being analyzed at ambient temperature. Bacterial inhibition was directly proportional to the number of NPs used, and 40–96% inhibition was observed at concentrations of 10–60 μg/mL. However, no effective inhibition of bacteria was observed above (60–80 μg/mL) the concentration of PtNPs, as illustrated in Figure 16A,B. The inhibitory efficacy of PtNPs is reduced at higher concentrations due to particle aggregation, which increases particle size and reduces the accessible surface area for nanoparticles [97].

#### 3.8.3. Antioxidant Activity

**Figure 17:**
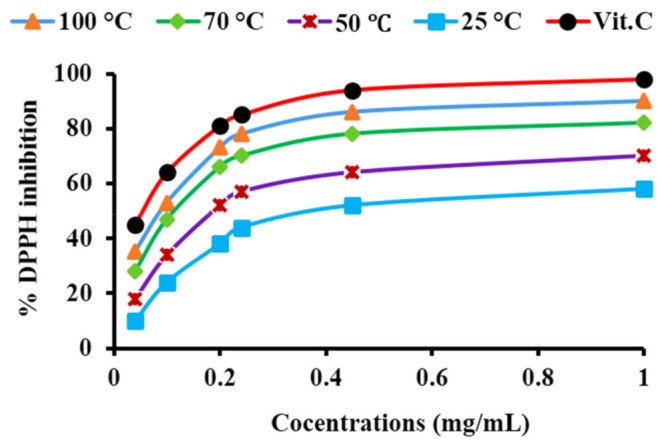
DPPH scavenging efficacy of Vit-C and PtNPs synthesized at 25 °C, 50 °C, 70 °C and 100 °C, respectively.

The antioxidant activity of PtNPs was determined by converting DPPH from a radical to a stable form. Vitamin-C (Ascorbic acid) was used as standard. DPPH is a stable radical capable of receiving electrons from PtNPs [98]. Figure 17 represents the DPPH activity of PtNPs synthesized at four different optimized conditions (i.e., 25 °C, 50 °C, 70 °C, and 100 °C). The results showed that PtNPs synthesized at 100 °C had the highest antioxidant activity (i.e., 88%) as compared to PtNPs synthesized at 70 °C, 50 °C, and 25 °C (76%, 69%, 56%), respectively. The size, morphology, and concentration of PtNPs, as well as the synergic effect and the presence of acid phosphatase on their surface, all contribute to the high efficiency of PtNPs [73]. Moreover, the radical conversion efficacy of PtNPs increased with increasing concentrations of nanoparticles. As a result, the strong antioxidant activity of PtNPs also confirms their biocompatibility and functionality in the living environment in terms of trapping oxidants to strengthen the body’s defense system [99].

## 4. Conclusions

In this study, PtNPs were successfully synthesized by an innovative, efficient, and cost-effective green synthetic approach using acid phosphatase of *Cichorium intybus* seeds at four different optimized conditions (i.e., 25 °C, 50 °C, 70 °C, and 100 °C). In addition, UV–vis, XRD, SEM, and HRTEM analysis confirmed the formation of crystalline monodispersed spherical PtNPs with an average size of 1–7 nm. The acid phosphatase mediated PtNPs exhibited remarkable photocatalytic activity with 98% degradation efficiency of MB and also followed pseudo-first-order kinetics. The results demonstrated that PtNPs can be used as an efficient catalyst for the degradation of organic pollutants such as MB dye in the presence of visible light irradiations. The biomorphic PtNPs were also tested for their antibacterial activity. The obtained results prove that acid phosphatase-mediated PtNPs are highly active against both Gram-positive (*S. aureus*) and Gram-negative (*E. coli*) bacteria. PtNPs were also highlighted for their potent application as the best antioxidant, with a DPPH radical scavenging activity of 88% at 1 mg/mL. The results demonstrated that the green synthesis process we followed can be used as a novel route for synthesizing PtNPs with applicability in various fields. Particularly, the superior photocatalytic and antimicrobial properties of PtNPs disclosed their potential use in food bioscience, biotechnology, biomedical and environmental applications.

## Figures and Tables

**Table 1:** Comparison between the catalytic degradation efficiency of ACP-PtNPs and other green synthesized PtNPs that were reported in other studies against MB.

Catalyst	MB Concentration	Degradation Efficiency (%)	Time (min)	Reference
PtNPs	4 mL	100	15	[70]
At-PtNPs	100 PPm	100	5	[71]
PtNPs	1.5 mL	100	15	[54]
SA-PtNPs	2 mL	39.87	1	[72]
PtNPs	500 mL	90.28	50	[73]
ACP-PtNPs	80 mL	99	28	[This work]

**Table 2:** Antibacterial activities of PtNPs synthesized at optimized conditions and standard drug against *E. coli* and *S. aureus* both in visible light and dark conditions.

Sample	Zone of Inhibition (mm)			
	Light		Dark	
	*E. coli*	*S. aureus*	*E. coli*	*S. aureus*
Standard	33	31	27	26
PtNPs (100 °C)	31	30	25	22
PtNPs (70 °C)	26	24	19	18
PtNPs (50 °C)	18	17	15	13
PtNPs (25 °C)	13	12	9	7

**Table 3:** Comparison between the antibacterial efficiency of PtNPs synthesized in the present study and other AgNPs.

Sample	Concentration	Bacterial Strain	Zone of Inhibition (mm)	Reference
AgNPs	75 µL	*E. coli/S. aureus*	18.5/14.9	[88]
AgNPs	20 µL	*E. coli/S. aureus*	14/13	[89]
AgNPs	2 µL	*E. coli/S. aureus*	8.8/8.5	[90]
AgNPs	20 µL	*E. coli/S. aureus*	n.a/n.a	[91]
AgNPs	100 µL	*E. coli/S. aureus*	8.39/8.54	[92]
PtNPs	50 µL	*E. coli/S. aureus*	31/30	[This work]

n.a = no activity at tested concentration.

**Table 4:** MIC of optimized PtNPs against *E. coli* and *S. aureus* bacteria.

Bacteria	PtNPs (µg/mL)			
	100 °C	70 °C	50 °C	25 °C
*E. coli*	10	20	20	30
*S. aureus*	10	20	20	30

## Data Availability

Not applicable.
